# Myocardial fibrosis in severe asymptomatic versus symptomatic aortic stenosis: A cardiac magnetic resonance cross-sectional study

**DOI:** 10.1007/s10554-025-03519-2

**Published:** 2025-09-18

**Authors:** Katrine Aagaard Myhr, Liv Andrés-Jensen, Bjørn Strøier Larsen, Charlotte Burup Kristensen, Nana Køber, Susanne Glasius Tischer, Lars Lindholm Sørensen, Søren Skøtt Schmiegelov, Jesper James Linde, Niels Grove Vejlstrup, Jordi Sanchez Dahl, Lars Køber, Redi Pecini

**Affiliations:** 1 Department of Cardiology, The Heart Centre, Copenhagen University Hospital – Rigshospitalet, Inge Lehmanns Vej 7, 2100 Copenhagen, Denmark; 2 Department of Pediatric Hematology/Oncology, Juliane Marie Centre, Copenhagen University Hospital – Rigshospitalet, Copenhagen, Denmark; 3 Copenhagen Oncology Research Laboratory (Bonkolab), Juliane Marie Centre, Copenhagen University Hospital – Rigshospitalet, Copenhagen, Denmark; 4 Department of Cardiology, Copenhagen University Hospital – Bispebjerg and Frederiksberg, Copenhagen, Denmark; 5 Department of Cardiology, Copenhagen University Hospital – Amager and Hvidovre, Copenhagen, Denmark; 6 Department of Cardiology, Zealand University Hospital, Roskilde, Denmark; 7 Department of Cardiology, Odense University Hospital, Odense, Denmark

**Keywords:** Aortic valve stenosis, Myocardial fibrosis, Cardiac magnetic resonance, Native T1 mapping, ECV mapping, Late gadolinium enhancement

## Abstract

Purpose The degree of myocardial fibrosis may guide the timing of aortic valve replacement in patients with severe asymptomatic aortic valve stenosis (AS). We aimed to examine the prevalence and degree of myocardial fibrosis in severe asymptomatic AS compared with severe symptomatic AS. Methods We prospectively included 42 patients with severe asymptomatic AS and 80 patients with severe symptomatic AS, all with a left ventricular ejection fraction ≥ 50% and no ischemic heart disease. All patients underwent a standard cardiac magnetic resonance examination, including native T1 mapping. Gadolinium contrast was administered to evaluate extracellular volume fraction (ECV%) and late gadolinium enhancement (LGE). Results There were no differences in myocardial fibrosis markers when comparing symptomatic with asymptomatic patients and adjusting for sex, age, and aortic peak velocity, including native T1 (mean difference [MD] -1 ms; 95% confidence interval [CI] -11 to 10; *p* = 0.90), ECV% (MD 0.2%; 95% CI -1 to 1; *p* = 0.65), LGE prevalence (odd ratio [OR] 1.2; 95% CI 0.4 to 3.3; *p* = 0.72), and a combined binary high- or low-grade fibrosis parameter (OR 1.1; 95% CI 0.3 to 4.0; *p* = 0.94). Conclusion Patients with severe asymptomatic AS exhibited similar severity of myocardial fibrosis markers as those with severe symptomatic AS, suggesting that substantial myocardial damage precedes symptom development.

**Graphical abstract:**

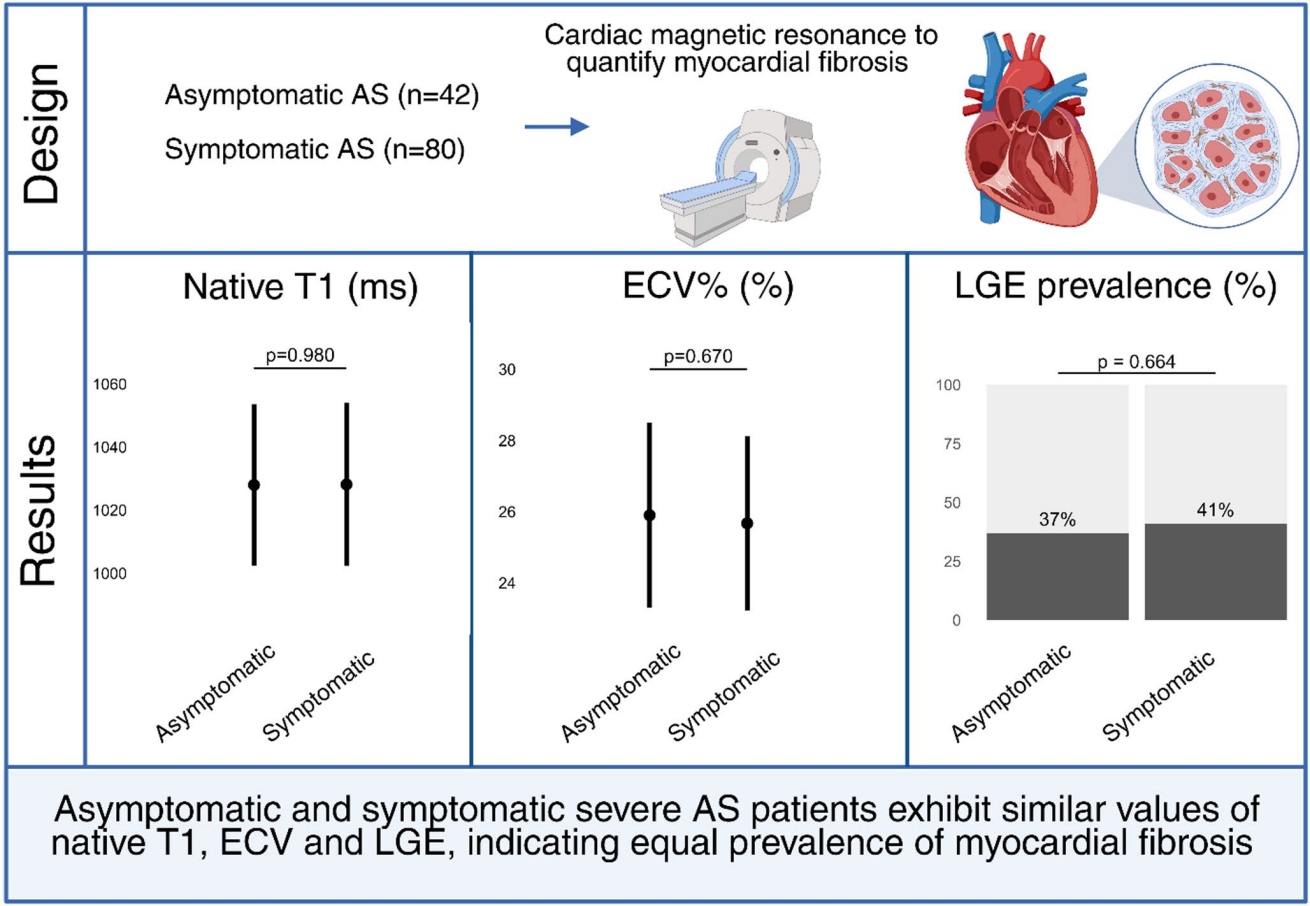
 AS, aortic valve stenosis; ECV, extracellular volume; LGE, late gadolinium enhancement Created with BioRender.

**Supplementary Information:**

The online version contains supplementary material available at 10.1007/s10554-025-03519-2.

## Introduction

Timely aortic valve replacement (AVR) in patients with aortic valve stenosis (AS) is key to improve prognosis. Although symptom development is currently considered a class I indication for AVR [1], recent small trials have suggested a survival advantage of earlier intervention [2–4]. However, asymptomatic patients constitute a heterogeneous group, necessitating better markers to identify those at highest risk.

Myocardial fibrosis has been proposed as a marker to guide the timing of AVR in asymptomatic AS [5]. Chronic pressure overload of the left ventricle (LV) leads to progressive compensatory hypertrophy along with myocardial interstitial fibrosis [6]. Eventually, this process results in diastolic and systolic dysfunction, culminating in heart failure [7–9]. Native T1 and extracellular volume (ECV) mapping are non-invasive cardiac magnetic resonance (CMR) methods to estimate the burden of diffuse fibrosis and have been shown to correlate with histology and stenosis severity in AS [10–12]. Additionally, late gadolinium enhancement (LGE) by CMR detects irreversible replacement fibrosis, which is considered to appear in the later stages of the disease. At the time of AVR, approximately 20–50% of patients with AS exhibit non-infarct LGE [13–15]. All three markers of myocardial fibrosis have independently been associated with impaired prognosis in AS [16, 17], suggesting that detection of myocardial fibrosis at an earlier stage may be valuable in the selection of patients for timely intervention. Despite these insights, the extent and prevalence of myocardial fibrosis in patients with severe asymptomatic AS remain understudied.

In this cross-sectional CMR study, our primary aim was to compare native T1, ECV fraction (ECV%), and LGE prevalence between patients with severe asymptomatic and symptomatic AS with a left ventricular ejection fraction (LVEF) ≥ 50% and without ischemic heart disease. Secondarily, we aimed to compare LV volumes and function between the two groups.

## Methods

### Study population

Patients with severe asymptomatic and symptomatic AS were included prospectively between November 2020 and August 2024 in Denmark as part of the two studies: the *Effects of Aortic Valve Replacement on Myocardial T1 values in Severe Aortic Valve Stenosis (FIBROTIC)* (NCT05404100) and the *Danish National Randomized Study on Early Aortic Valve Replacement in Patients With Asymptomatic Severe Aortic Stenosis (DANAVR)* (NCT03972644). Symptomatic patients were identified at the Heart Team conferences for AVR referrals, and asymptomatic patients were included in outpatient clinics and did not meet AVR guideline criteria. Patients were classified as asymptomatic based on a comprehensive assessment by a senior physician, which included an exercise test in 62% of cases. The definition of severe AS differed slightly between studies: In FIBROTIC, which included both symptomatic and asymptomatic patients, severe AS was defined by aortic peak velocity (Vmax) > 4 m/s and/or aortic mean gradient > 40 mmHg, while in DANAVR, which included only asymptomatic patients, severe AS was defined by an aortic valve area ≤ 1 cm^2^ and Vmax ≥ 3.5 m/s. Additionally, DANAVR inclusion criteria included signs of elevated filling pressure or impaired longitudinal LV function, evidenced by a left atrial volume index > 34 ml/m^2^, an E/e’ ratio > 13, NT-proBNP levels three times higher than the upper limit for age and sex, or a global longitudinal strain (GLS) ≥ −15%. General exclusion criteria were: LVEF < 50%, ischemic heart disease defined by a history of myocardial infarction and/or previous or planned primary coronary intervention or coronary artery bypass grafting, moderate or severe left-sided valvular heart disease other than AS, permanent atrial fibrillation, pregnancy, pacemaker or implantable cardioverter defibrillator, and severe claustrophobia. All participants included in FIBROTIC underwent CMR. All participants in DANAVR recruited in the eastern part of Denmark and who did not fulfill any of the above exclusion criteria were invited to participate in this CMR study. Consequently, DANAVR patients with known ischemic heart disease were excluded.

The studies involved were conducted in accordance with the Declaration of Helsinki and approved by the Regional Ethics Committee for the Capital Region and Southern Region in Denmark (H-20029458, S-20190006). Written informed consent was obtained from all participants.

### Clinical data

The medical history of all participants was obtained through questionnaires and medical records. Baseline characteristics of AS patients included assessment of symptomatic status (i.e., New York Heart Association functional class, Canadian Cardiovascular Society grading of angina pectoris with a class 0 for no angina, and history of syncope) and registration of comorbidities and medications. The EuroSCORE II [18] was calculated for all patients. The score includes a categorization of the expected procedure, which therefore had to be assumed for the asymptomatic patients in DANAVR randomized to watchful waiting. As none of these had an ascending aorta > 5 cm on echocardiography, an isolated AVR was chosen. Echocardiographic and CT data were collected from clinical examinations. An aortic valve calcium score ratio was calculated as aortic valve calcium score divided by 1200 for females and 2000 for males. All hematocrit values were measured on the same day as the CMR examination. Blood pressures were measured in the supine position following the CMR examination.

### Cardiac magnetic resonance

All participants underwent a CMR examination on the same Siemens MAGNETOM Aera 1.5 Tesla Scanner (Syngo MR E11 software) without upgrading either software or hardware during the study period. Standard cine images of the LV were acquired, and native T1 mapping was performed using the modified Look-Locker inversion recovery (MOLLI) 5(3)3 acquisition scheme with three short-axis subsets of the LV, as previously described [19]. Patients received intravenous Gadobutrol (Gadovist^®^, Bayer, Germany) for LGE assessment and ECV% calculation. The administered dose varied by estimated glomerular filtration rate (eGFR): 0.15 mmol/kg for eGFR > 59 mL/min/1.73 m^2^, 0.1 mmol/kg for eGFR 30–59 mL/min/1.73 m^2^, and no contrast for eGFR < 30 mL/min/1.73 m^2^. Post-contrast T1 mapping images were acquired 10 min following administration. LGE images were acquired approximately 12 min post-contrast, including short-axis images of the LV and four-, two-, and three-chamber views. In cases of LGE, an orthogonal view was acquired for verification. The inversion time was continually adjusted for the correct nulling of the myocardium.

CMR image analysis was performed using Circle CVI 42^®^, version 5.13.5, Calgary, Canada. All examinations were pseudo-anonymized and analyzed by K.A.M. LV volumes, mass, and function were assessed as previously described [19]. LV hypertrophy was defined from sex- and age-based reference ranges [20]. Global native T1 mapping analysis was performed on motion-corrected MyoMaps as previously described [19]. The test-retest reproducibility of native T1 mapping has previously been assessed by our group and showed 95% limits of agreement of ± 26 ms [21]. ECV% was defined as ECV% = (1-hematocrit) x (Δ(1/T1_myocardium_)/Δ(1/T1_blood_)*100). Global ECV% mapping analysis was performed using pre- and post-contrast motion-corrected MyoMaps. Areas of LGE were not excluded on T1 and ECV maps. Hinge-point LGE was not defined as LGE-positive as it has limited prognostic value and is considered a non-specific finding both seen in healthy individuals and various cardiac conditions [22, 23]. Endocardial LGE was classified as ischemic, and mid-wall or epicardial LGE was classified as non-ischemic. LGE volume was quantified as percentage of total LV myocardial volume using the full width half maximum (FWHM) method [24]. GLS analysis was performed on long-axis views, with automatic delineation of the LV endo- and epicardium and subsequent generation of peak systolic GLS.

LGE and ECV mapping images were feasible for analysis in 107 patients (88%) and 103 patients (84%), respectively. The remaining examinations were not possible due to incomplete scans because of claustrophobia or back pain, difficulties with intravenous access, patients declining to receive gadolinium contrast, poor image quality, image post-processing issues, or missing hematocrit data.

### Statistical analysis

Normally distributed data are presented as mean ± standard deviation, non-normally distributed as median (interquartile range), and categorical data as count (percentage). Data normality was evaluated using histograms and Q–Q plots. P-values ≤ 0.05 were considered statistically significant. In cases of missing data, the total number of observations for a given variable is provided in square brackets in Tables 1 and 2. Baseline characteristics were compared using Student’s *t*-test for normally distributed data, the Wilcoxon signed-rank test for non-normally distributed data, and the chi-square test with Monte Carlo simulation for categorical data. Correlations between CMR fibrosis markers and NT-proBNP levels were calculated using Spearman’s rank correlation coefficient (*ρ*). Comparison of CMR parameters between asymptomatic and symptomatic AS patients was done using multivariable linear or logistic regression analyses adjusted for sex, age, and Vmax. Based on the approach in a previous study [15], native T1, ECV%, and LGE prevalence were combined into a single binary high- or low-grade variable of myocardial fibrosis. A combination of LGE positivity, an ECV% above the sex-specific median value, and a native T1 above the sex-specific median value defined high-grade fibrosis. Patients not meeting these criteria were classified as having low-grade fibrosis. The median value of native T1 was 1026 ms for males and 1020 ms for females, and the median value of ECV% was 25.6% for males and 25.3% for females. Due to the unequal proportion of bicuspid aortic valvular morphology between symptomatic and asymptomatic patients, post hoc subgroup analyses were performed on those with a tricuspid aortic valve using multivariable linear and logistic regression analyses adjusted for sex, age, and Vmax. Further, a post hoc chi-square test with Monte Carlo simulation was performed to compare the prevalence of LGE between males and females. Statistical analyses and the creation of Figs. 2 and 3 were performed using R Statistical Software (v4.4.1; R Core Team 2024).

## Results

### Patient characteristics

Baseline characteristics are presented in Table 1. The study initially included 42 patients with asymptomatic AS and 86 patients with symptomatic AS. However, six symptomatic patients were excluded due to claustrophobia, leaving 42 asymptomatic and 80 symptomatic patients. Of the 42 asymptomatic patients, 6 were included from the FIBROTIC study. None of the patients had a confirmed or suspected coexisting cardiomyopathy, including cardiac amyloidosis, hypertrophic cardiomyopathy, cardiac sarcoidosis, Fabry disease, other cardiac storage disorders, or myocarditis.

**Table 1:** Baseline characteristics of patients with asymptomatic (*n* = 42) and symptomatic (80) severe aortic stenosis

Characteristic	Asymptomatic AS (*n* = 42)	Symptomatic AS (*n* = 80)	*p*-value
Patient characteristics			
Age, years	73 ± 6	69 ± 11	0.01
Male sex, n (%)	24 (57)	59 (74)	0.08
BMI, kg/m^2^	27 ± 4	27 ± 4	0.87
BSA, m^2^	2.1 ± 0.6	2.0 ± 0.4	0.87
Heart rate, bpm	68 ± 11 *[n = 41]*	68 ± 12 *[n = 79]*	0.97
Systolic blood pressure, mmHg	143 ± 13 *[n = 40]*	138 ± 15 *[n = 72]*	0.04
Diastolic blood pressure, mmHg	81 ± 10 *[n = 40]*	78 ± 9 *[n = 72]*	0.14
Disease characteristics			
Bicuspid aortic valve, n (%)	6 (14)	38 (48)	< 0.001
Aortic stenosis subtype^a^, n (%)			
High gradient	35 (83)	76 (95)	0.02
Low flow, low gradient	0 (0)	1 (1)	
Normal flow, low gradient	7 (17)	3 (4)	
Very severe AS^b^, n (%)	7 (17)	30 (38)	0.02
Aortic valve peak velocity, m/s	4.3 (4.0–4.6)	4.6 (4.2–4.9)	0.003
Aortic valve mean gradient, mmHg	48 (42–57)	54 (45–64)	0.02
Aortic valve area VTI, cm^2^	0.8 (0.7–0.9)	0.8 (0.7–0.9) *[n = 78]*	0.35
Left atrial volume index, ml/m^2^	34 ± 12 *[n = 41]*	35 ± 11 *[n = 61]*	0.50
E/e’ ratio	13 ± 4 *[n = 39]*	12 ± 5 *[n = 54]*	0.42
Aortic valve calcium score, AU	3234 (2796–4392) *[n = 14]*	3586 (2493–5188) *[n = 68]*	0.71
Aortic valve calcium score ratio^c^	2.1 (1.5–2.7) *[n = 14]*	2.0 (1.5–2.9) *[n = 68]*	0.59
Coronary artery calcium score, AU	180 (69–618) *[n = 14]*	224 (42–592) *[n = 68]*	0.10
NYHA class n (%)			
I	42 (100)	8 (10)	< 0.001
II	0 (0)	58 (73)	
III	0 (0)	14 (17)	
IV	0 (0)	0 (0)	
CCS, n (%) 0 1 2 3 4	42 (100) 0 (0) 0 (0) 0 (0) 0 (0)	40 (50) 25 (31) 14 (18) 1 (1) 0 (0)	< 0.001
Syncope, n (%)	0 (0)	9 (11)	0.03
EuroSCORE II	1.03 (0.84–1.16)	1.12 (0.79–1.50)	0.46
Comorbidities and risk factors			
Hypertension, n (%)^d^	32 (76)	47 (59)	0.07
Diabetes mellitus, n (%)^e^	6 (14)	12 (15)	1.00
Smoking, n (%) Current Former Never	*[n = 41]* 2 (5) 16 (39) 23 (56)	*[n = 79]* 4 (5) 41 (52) 34 (43)	0.40
Atrial fibrillation, n (%)	1 (2)	11 (14)	0.05
Stroke, n (%)	4 (10)	11 (14)	0.58
Sleep apnea, n (%)	1 (2)	7 (9)	0.23
Peripheral artery disease, n (%)	1 (2)	2 (3)	1.00
Cancer, n (%)^f^	2 (5)	6 (8)	0.71
COPD, n (%)	1 (2)	5 (6)	0.45
Medications			
Antihypertensives, n (%)	31 (74)	44 (55)	0.05
Lipid-lowering therapy, n (%)	11 (26)	45 (56)	0.07
Anti-platelet therapy, n (%)	12 (29)	23 (29)	1.00
Anticoagulants, n (%)	2 (5)	11 (14)	0.22
Antidiabetics, n (%)	4 (10)	11 (14)	0.57
Biochemistry			
Troponin T, ng/L	15 (11–21) *[n = 30]*	14 (10–18) *[n = 69]*	0.51
NT-proBNP, pmol/L	39 (26–87) *[n = 40]*	48 (24–96) *[n = 77]*	0.86
Creatinine, µmol/L	80 ± 18 *[n = 41]*	83 ± 17 *[n = 79]*	0.49
eGFR, ml/min/1.73 m^2^	75 ± 12 *[n = 41]*	78 ± 18 *[n = 79]*	0.32
Hematocrit	0.4 ± 0.04 *[n = 39]*	0.4 ± 0.05 *[n = 73]*	0.64
HbA1c, mmol/mol	38 (35–40) *[n = 38]*	38 (35–41) *[n = 74]*	0.55
Total cholesterol, mmol/L	4.3 (3.8–4.9) *[n = 40]*	4.5 (3.8–5.2) *[n = 74]*	0.67
LDL cholesterol, mmol/L	2.2 (2.0–2.9) *[n = 40]*	2.6 (1.9–3.1) *[n = 75]*	0.34
HDL cholesterol, mmol/L	1.4 (1.2–1.7) *[n = 40]*	1.5 (1.2–1.9) *[n = 75]*	0.80
Triglycerides, mmol/L	1.4 (1.1–1.7) *[n = 40]*	1.3 (0.9–1.8) *[n = 73]*	0.36

Values are mean ± SD, median (interquartile range), or count (percentage). In case of missing observations, the total number of observations is presented in square brackets

BMI, body mass index; BSA, body surface area; CCS, Canadian Cardiovascular Society grading of angina pectoris; COPD, chronic obstructive pulmonary disease; eGFR, estimated glomerular filtration rate; E/e’ ratio, the ratio of early diastolic transmitral flow velocity to early diastolic mitral annular velocity; HbA1c, hemoglobin A1c; HDL, high-density lipoprotein; LDL, low-density lipoprotein; NT-proBNP, N-terminal pro–B-type natriuretic peptide; NYHA, New York Heart Association Functional Classification of heart failure; VTI, velocity time integral

^a^ Aortic stenosis subtypes: High-gradient (aortic mean gradient ≥ 40 mmHg); low-flow, low gradient (aortic mean gradient < 40 mmHg, aortic valve area ≤ 1 cm^2^, LVEF ≥ 50%, stroke volume index ≤ 35 mL/m^2^); normal-flow, low-gradient (aortic mean gradient < 40 mmHg, aortic valve area ≤ 1 cm^2^, LVEF ≥ 50%, stroke volume index > 35 mL/m^2^

^b^ Very severe aortic stenosis: aortic peak velocity > 5 m/s or aortic mean gradient ≥ 60 mmHg

^c^ Aortic valve calcium score ratio: Aortic valve calcium score divided by 1200 for females and 2000 for males

^d^ Hypertension: Known or use of anti-hypertensive medications (β-blockers, angiotensin-converting enzyme inhibitors, angiotensin receptor blockers, calcium inhibitors, and diuretics)

^e^ Diabetes mellitus: Type 1, type 2, or HbA1c > 48 mmol/mol

^f^ Cancer: Current or former types of cancers treated with systemic therapy and/or chest radiotherapy

### Cardiac magnetic resonance characteristics

**Fig. 1:**
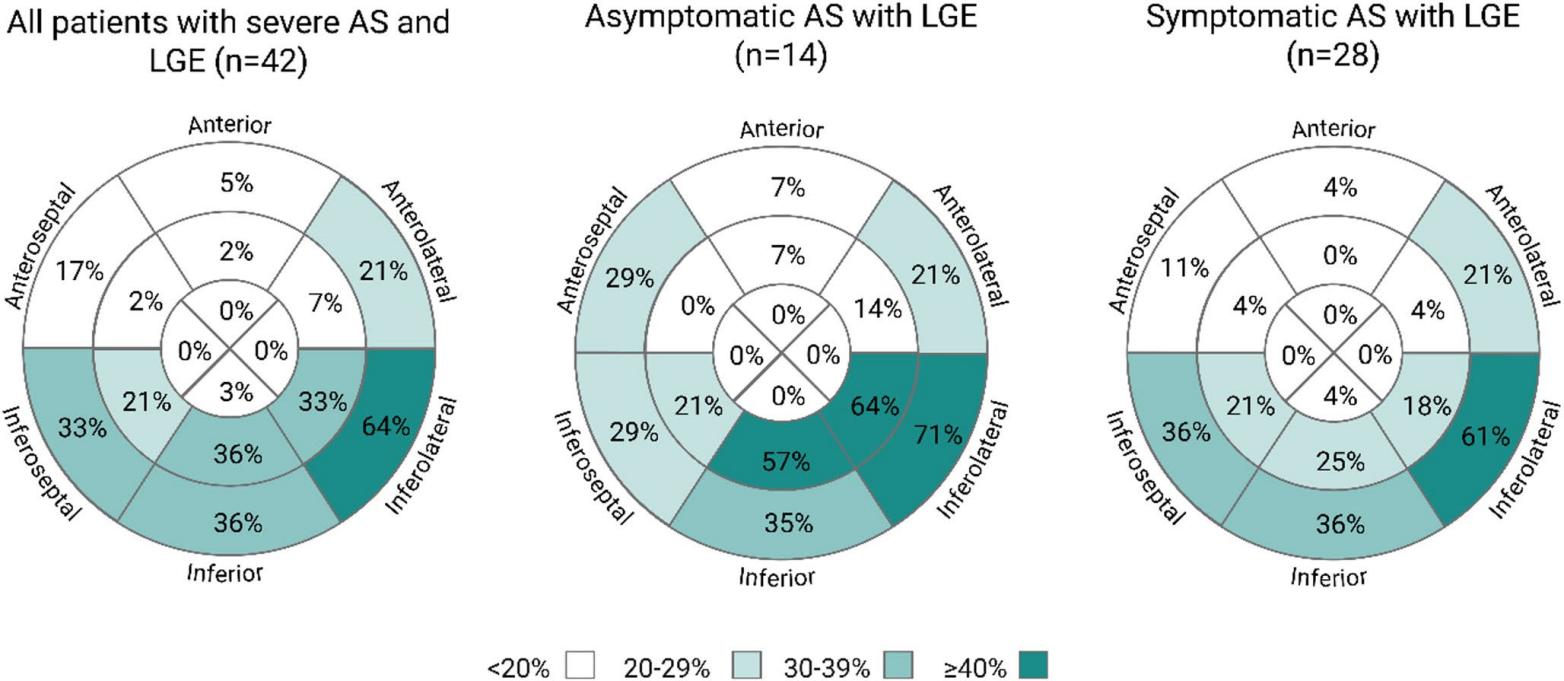
American Heart Association 16-segment model illustrating the prevalence (%) of late gadolinium enhancement (LGE) in individual myocardial segments among all patients with aortic valve stenosis (AS) and positive LGE (left), as well as separately for asymptomatic (middle) and symptomatic (right) patients. Created with BioRender

CMR parameters of patients with asymptomatic and symptomatic AS are presented in Table 2. The overall prevalence of LGE in AS patients was 39%, with non-ischemic LGE being more common than ischemic LGE (88% vs. 12%, *p* < 0.001). LGE was primarily located in the basal and mid inferoseptal, inferior, and inferolateral segments (Fig. 1). Male AS patients had a higher LGE prevalence than females (53% vs. 9%; X^2^ = 19; *p* < 0.001). NT-proBNP levels correlated with native T1 (*ρ* = 0.50, *p* < 0.01) and ECV% (*ρ* = 0.32, *p* < 0.01), but not with LGE prevalence (*ρ* = 0.18, *p* = 0.06).

**Table 2:** Baseline cardiac magnetic resonance parameters and comparison of patients with asymptomatic (reference) (*n* = 42) and symptomatic (*n* = 80) severe aortic stenosis using multivariable linear and logistic regression analyses adjusted for sex, age, and peak aortic velocity

Parameter	Asymptomatic AS (*n* = 42)	Symptomatic AS (*n* = 80)	Comparison: β or OR (95% CI)		*p*-value
Left ventricular geometry and function					
LVEDVi, ml/m^2^	74 ± 19	77 ± 15 *[n = 79]*	1 (−5 to 6)	0.82	
LVESVi, ml/m^2^	22 ± 11	25 ± 9 *[n = 79]*	3 (−1 to 7)	0.12	
LVSVi, ml/m^2^	51 ± 12	52 ± 12 *[n = 79]*	−2 (−7 to 2)	0.31	
LVMi, g/m^2^	77 ± 19	79 ± 18 *[n = 79]*	−3 (−9 to 4)	0.39	
LV hypertrophy, n (%)	17 (40)	27 (34) *[n = 79]*	0.5 (0.2 to 1.3)	0.16	
LVEF, %	70 ± 9	68 ± 9 *[n = 79]*	−4 (−8 to −1)	0.02	
GLS, %	−17 ± 2 *[n = 41]*	−16 ± 2 *[n = 78]*	1 (−0.2 to 1.5)	0.15	
Myocardial tissue characterization					
Native T1, ms	1028 ± 26	1028 ± 26	−1 (−11 to 10)	0.90	
ECV%, %	26 ± 3 *[n = 36]*	26 ± 2 *[n = 67]*	0.2 (−0.8 to 1.3)	0.65	
LGE positive, n (%)	14 (37) *[n = 38]*	28 (41) *[n = 69]*	1.2 (0.4 to 3.3)	0.72	
LGE pattern, n (%) Ischemic Non-ischemic	0 (0) 14 (100)	5 (18) 23 (82)	0.18 (0.001 to 2.0)	0.18	
LGE volume percentage, %	2.1 (0.9–3.8)	2.0 (0.8–3.5)	−0.4 (−1.7 to 0.9)	0.50	
Myocardial fibrosis grade, n (%)^a^ High-grade Low-grade	*[n = 35]* 5 (14) 30 (86)	*[n = 64]* 9 (14) 55 (86)	1.1 (0.3 to 4.0)	0.94	

Baseline values are mean ± SD, median (interquartile range), or count (percentage). In case of missing observations, the total number of observations is presented in square brackets. Linear and logistic regression analyses are presented with β-values or odds ratios and 95% confidence intervals, respectively. Asymptomatic patients were used as reference

ECV%, extracellular volume; GLS, global longitudinal strain; LGE, late gadolinium enhancement; LV hypertrophy, left ventricular hypertrophy; LVEDVi, left ventricular end-diastolic volume index; LVSVi, left ventricular end-systolic volume index; LVEF, left ventricular ejection fraction; LVMI, left ventricular mass index; LVSVi, left ventricular stroke-volume index

^a^ Myocardial fibrosis grade: Native T1, ECV%, and LGE prevalence combined in a single high-grade or low-grade myocardial fibrosis parameter

### Symptomatic versus asymptomatic aortic stenosis

**Fig. 2:**
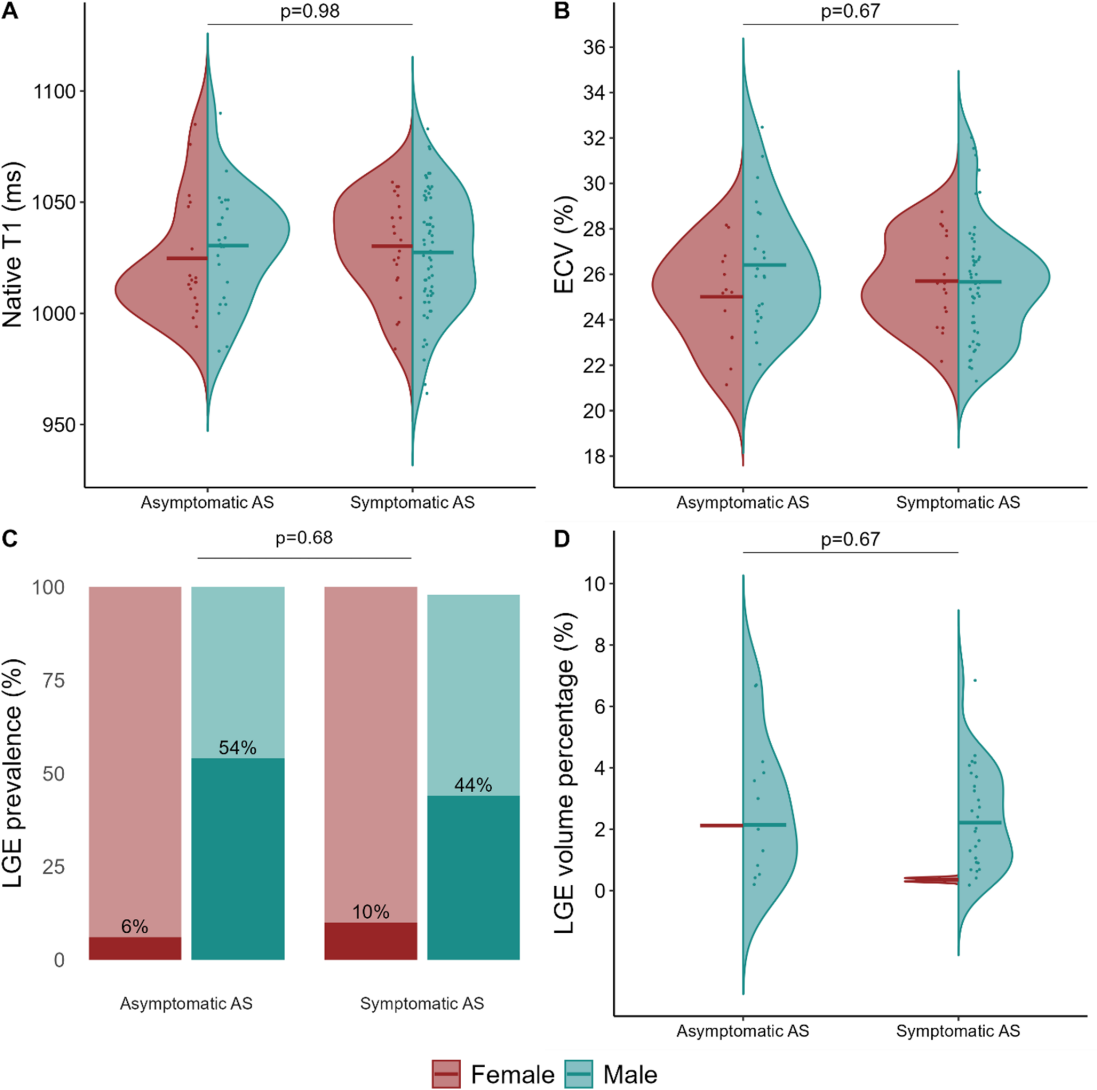
Comparison of native T1 (A), extracellular volume fraction (ECV%) (B), late gadolinium enhancement (LGE) prevalence (C), and LGE volume percentage (D) in patients with severe asymptomatic (*n* = 42) and symptomatic (*n* = 80) aortic stenosis. Continuous variables are presented as violin plots (A, B, and D) and stratified in females (red) and males (blue). Dots represent individual observations, and horizontal lines represent mean or median values, as appropriate. LGE prevalence is presented as a bar chart (C), stratified by sex

**Fig. 3:**
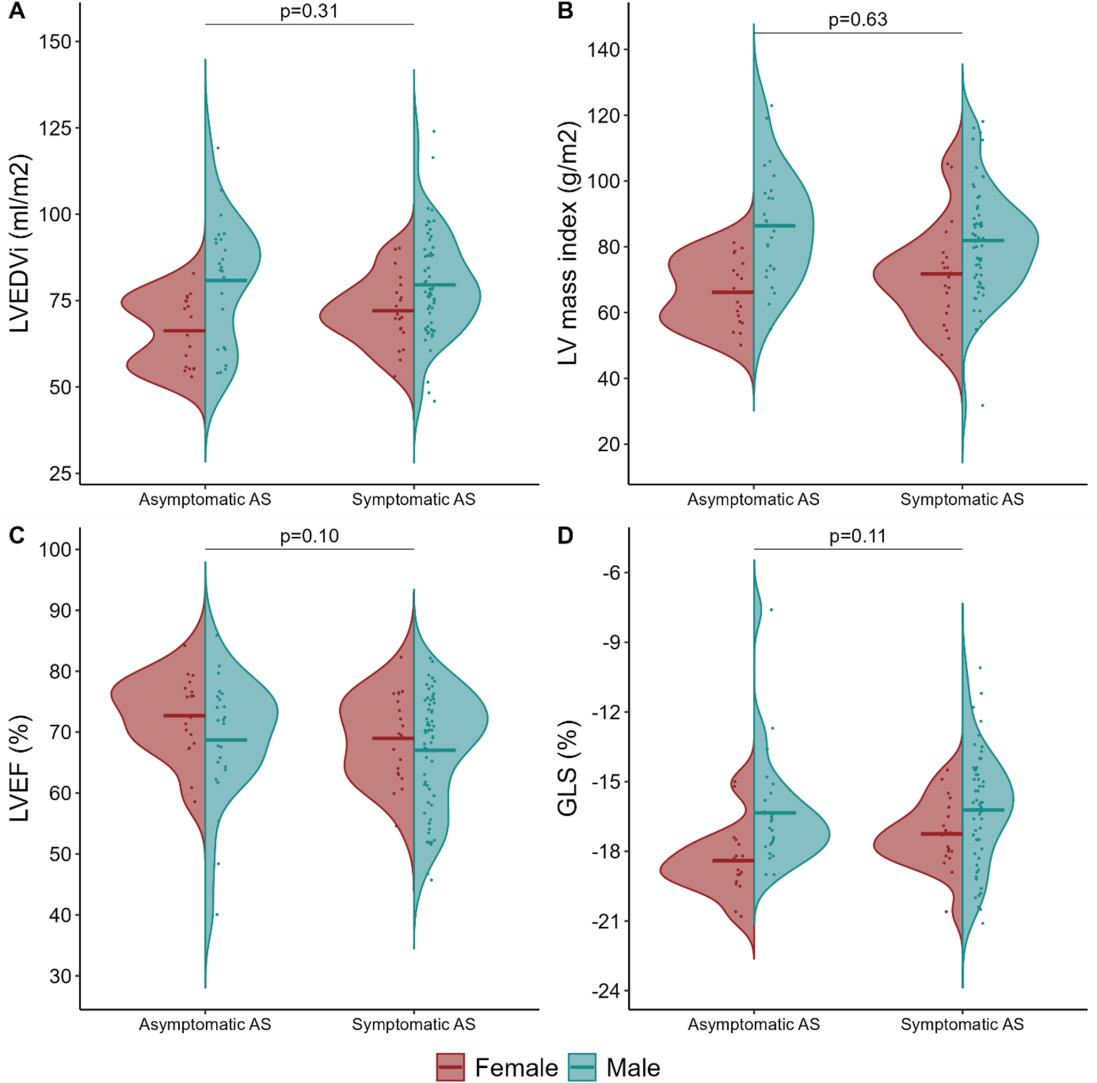
Comparison of left ventricular end-diastolic volume index (LVEDVi) (A), left ventricular mass index (LV mass index) (B), left ventricular ejection fraction (LVEF) (C), and global longitudinal strain (GLS) (D) in patients with severe asymptomatic (*n* = 42) and symptomatic (*n* = 80) aortic stenosis. The distributions are presented as violin plots stratified in females (red) and males (blue). Dots represent individual observations, and horizontal lines represent mean values

Figure 2 illustrates the distribution and comparisons of myocardial fibrosis markers between patients with severe asymptomatic and symptomatic AS. There were no differences in native T1, ECV%, LGE prevalence, or LGE volume percentage between asymptomatic and symptomatic patients (all *p* > 0.05). These findings were confirmed in analyses adjusted for sex, age, and Vmax (Table 2). When native T1, ECV%, and LGE prevalence were combined into a single binary variable representing high-grade or low-grade myocardial fibrosis, no difference was found between the asymptomatic and symptomatic patients (Table 2). Additionally, no differences were observed in LV volumes, LV mass, or GLS (Fig. 3; Table 2). However, symptomatic patients had a 4-percentage point lower LVEF compared with asymptomatic patients of the same sex, age, and stenosis severity (Table 2). Subgroup analyses were performed on those with a tricuspid aortic valve (42 symptomatic vs. 36 asymptomatic; mean age 76 vs. 75 years; 69 vs. 58% males) due to the difference in valvular morphology between asymptomatic and symptomatic patients. There were no differences in native T1 (β 2.8 ms; 95% CI −8 to 14; *p* = 0.62), ECV% (β 0.6; 95% CI −0.7 to 1.9; *p* = 0.37), LGE prevalence (OR 2.0; 95% CI 0.5 to 8.1; *p* = 0.30), or LGE volume percentage (β −0.3; 95% CI −1.7 to 1.2; *p* = 0.70) between asymptomatic and symptomatic patients. In sensitivity analyses, the results remained unchanged when excluding the asymptomatic patients enrolled from the FIBROTIC study (*n* = 6) (Supplemental Table S1). Likewise, excluding those with ischemic LGE (*n* = 5) did not alter the findings (Supplemental Table S2).

## Discussion

In this cross-sectional CMR study, patients with severe asymptomatic AS exhibited similar values of myocardial fibrosis parameters, including native T1, ECV%, and LGE prevalence, compared to patients with severe symptomatic AS. Our findings indicate that substantial myocardial damage precedes symptom development in severe AS, challenging the notion that surgery in asymptomatic patients may prevent advanced structural changes in the myocardium. This study is among the few that have evaluated the prevalence and degree of myocardial fibrosis in asymptomatic severe AS, despite its proposed role in guiding early intervention.

Our study found that irreversible replacement fibrosis assessed by LGE was equally prevalent in asymptomatic and symptomatic patients. This contrasts with a previous study (*n* = 169) reporting a higher prevalence of LGE in severe asymptomatic AS [25]. However, patients with ischemic heart disease were included, and the difference in LGE seemed driven by ischemic rather than non-ischemic fibrosis. The overall LGE prevalence of 39% in our study, with a predominance among males, aligns with previous studies examining patients with severe AS undergoing AVR [13–15, 26]. Although the LGE pattern was primarily non-ischemic, 18% of LGE-positive symptomatic patients exhibited an ischemic pattern despite the exclusion of patients with obstructive ischemic heart disease. The occurrence of ischemic LGE in AS patients without coronary artery disease has similarly been reported [14] and may be attributed to an oxygen supply-demand mismatch or microvascular disease. Further, ischemic LGE has been observed in a recent population-based study of individuals without coronary artery disease, and its presence was associated with diabetes [27]. In a small study by Bull et al. [10] of 22 severe asymptomatic and 24 severe symptomatic patients, native T1 values were higher in those who were symptomatic. This contrasts with our findings, most likely reflecting differences in comorbidities at baseline, as symptomatic patients were more likely to have diabetes and hypertension. Although no difference in native T1 was found between symptomatic and asymptomatic patients in our study, it is noteworthy that the mean value of 1028 ms in both groups exceeds the reference values observed in a healthy population who underwent native T1 mapping on the same scanner at our institution [19]. Further, the GLS values of −17% and − 16% in the asymptomatic and symptomatic patients were higher compared with a mean GLS of −19% found in the same healthy population (unpublished data). These findings support that these patients do have elevated levels of myocardial fibrosis. Previous studies have reported ECV% values around 27–28% [28–30] in patients with severe AS, compared with a mean ECV% of 25% in healthy participants [28], although ranges have overlapped. Our study found a mean ECV% of 26%, but without measurements in healthy participants, we cannot confirm that ECV% was above normal in our patients. Nevertheless, we believe our study is the first to directly compare ECV% between patients with asymptomatic and symptomatic severe AS.

Our findings raise a key question: if myocardial fibrosis is a primary driver of symptom development in AS, why did asymptomatic and symptomatic patients exhibit a comparable fibrosis burden? Several factors could account for this. Firstly, objectifying symptoms in an elderly population with competing comorbidities and various levels of physical fitness is highly challenging, and the classification into symptomatic and asymptomatic can be ambiguous. Secondly, the two populations may lie close on the spectrum of disease severity. In this study, parameters associated with symptomatic status, such as LV mass index and echocardiographic markers of LV filling pressure [9], did not differ between the asymptomatic and symptomatic patients. Neither did NT-proBNP, which has been shown to correlate with both AS severity and NYHA class in symptomatic patients [31]. However, most symptomatic patients belonged to NYHA class II, and there have been inconsistent results regarding the ability of natriuretic peptides to discriminate between asymptomatic and minimally symptomatic AS [32, 33]. Further, most of the asymptomatic patients were drawn from the DANAVR trial, meeting criteria for signs of elevated filling pressure on echocardiography. Considering that increased LV filling pressure in AS is believed to result from both LV hypertrophy and myocardial fibrosis, the asymptomatic patients in our study may have more advanced myocardial disease compared with the broader population of asymptomatic severe AS. However, signs of elevated filling pressure are common in patients with early-stage AS [34, 35], suggesting that the DANAVR cohort may not be narrowly preselected. Even if the two populations are close in disease severity, this comparison remains clinically essential, as current guidelines offer distinct recommendations based on symptom status. Finally, the onset of symptoms may not always align with the extent of myocardial remodeling and fibrosis, as other mechanisms, such as arrhythmias or microvascular ischemia, may precipitate symptoms. In addition, myocardial fibrosis may develop even before significant hemodynamic stenosis occurs, possibly related to comorbidities [36]. For instance, patients with diabetes mellitus often present with myocardial fibrosis, increased LV filling pressures, and diastolic dysfunction [37], and the coexistence of AS may lead to additional impairment [38]. Separating the underlying causes of fibrosis and heart failure symptoms in these patients is therefore highly challenging. The importance of comorbidities in patients with AS has recently been demonstrated in 9611 patients with mild AS [34]. In this study, 80% of patients had manifest cardiac damage assessed by echocardiography despite AS only being mild, most likely the consequence of coexisting comorbidities rather than AS per se. Further, individual differences in myocardial adaptation to pressure overload may explain why some patients with only moderate AS develop symptoms [39]. In summary, symptom burden in AS is multifactorial and may be discordant with stenosis severity and fibrosis extent. This may help to explain the similar fibrosis burden across symptom groups in our study.

Our findings corroborate the growing understanding that irreversible myocardial damage precedes symptom development and suggest that myocardial fibrosis alone does not account for the transition from asymptomatic to symptomatic status in severe AS. Given the established association between myocardial fibrosis and impaired clinical outcomes [16, 17], these findings underscore the need for a more comprehensive risk stratification in AS and investigation into the potential benefits of earlier intervention. In the Early TAVR trial (*n* = 901), transcatheter aortic valve replacement (TAVR) in severe asymptomatic AS improved clinical outcomes compared with clinical surveillance [40]. Conversely, the EVOLVED trial (*n* = 224) found no benefit of early intervention in patients with asymptomatic severe AS and concomitant LGE [41], raising the possibility that the opportunity to reverse myocardial decompensation is diminished once patients develop replacement fibrosis. In the TAVR UNLOAD trial (*n* = 178), early TAVR in patients with moderate AS and heart failure with reduced ejection fraction failed to improve clinical outcomes compared with conservative management [42], potentially influenced by a high burden of comorbidities and a predominance of ischemic cardiomyopathy. These findings underscore the challenges of separating valvular and non-valvular mechanisms behind myocardial dysfunction and symptom burden. Ongoing trials like the EXPAND TAVR II Pivotal trial (NCT05149755) and the PROGRESS trial (NCT04889872) will provide further insight into the effects of early intervention in moderate AS. While myocardial tissue characterization offers potential for enhancing risk stratification in AS, evidence is still limited. Its value as a tool to guide early surgery may be complicated by the potential influence of comorbidities, sex-related differences, and the overlap in reference ranges for native T1 and ECV%. Prospective studies are needed to determine whether incorporating these techniques into clinical practice improves patient outcomes. Finally, our post-hoc analyses revealed that LGE was more prevalent in males than in females. While the underlying mechanisms remain unclear, they may relate to sex-specific adaptive responses to pressure overload, hormonal influences, or variations in comorbidities. These findings underscore the importance of considering sex differences in clinical trials.

### Limitations

The limited sample size prevented matching on potential confounders such as sex, age, and comorbidities. Instead, we adjusted for sex, age, and Vmax. Since the asymptomatic patients were older, comorbidities could have influenced the results. Systolic blood pressure was higher among asymptomatic patients and might contribute to a higher fibrosis burden, especially in the mild and moderate stages of the disease. Further, we cannot exclude the possibility that the higher prevalence of a bicuspid valve among symptomatic patients may have influenced the results and affected the generalizability to the broader severe AS population. Due to the sample size, we were unable to account for valvular morphology in our analyses. However, our main findings remained consistent in subgroup analyses. In addition, we are currently exploring the association between myocardial fibrosis and the presence of either a bicuspid or tricuspid aortic valve in a separate study. Although all patients were classified as having severe AS, symptomatic patients showed slightly greater stenosis severity on echocardiography, and asymptomatic patients displayed a higher prevalence of normal-flow, low-gradient AS. While this limits comparability between groups, it may also reinforce that stenosis severity and the extent of myocardial fibrosis are not strictly linearly related. As a cross-sectional study, the design has inherent limitations. An extended follow-up including patients with mild and moderate AS may provide further insight into the progression of myocardial fibrosis and its association with symptom development and comorbidities. Further, the asymptomatic patients did not undergo systematic exercise tests. However, we wished this study to reflect standard clinical practice. It is important to note that native T1 and ECV% are not specific markers of myocardial fibrosis but are used as surrogates, and other tissue characteristics, such as edema, may affect both values. Further, recent histological correlation studies have not been able to confirm a correlation between fibrosis on histology and global ECV% measurements [15, 43].

## Conclusion

In this cross-sectional CMR study, patients with severe asymptomatic and symptomatic AS demonstrated comparable levels of myocardial fibrosis, suggesting that substantial myocardial damage is already present before symptom onset. The results highlight the complexity of disease progression in AS and support the need for improved risk stratification beyond symptom status.

## Supplementary Information

Below is the link to the electronic supplementary material.

Supplementary Material 1

## Data Availability

No datasets were generated or analysed during the current study.
