# Supplementary material for: Myocardial fibrosis in severe asymptomatic versus symptomatic aortic stenosis: A cardiac magnetic resonance cross-sectional study
Source: Int J Cardiovasc Imaging. 2025 Sep 18;41(11):2159–71. doi: 10.1007/s10554-025-03519-2 (PMC12628423; doi:10.1007/s10554-025-03519-2)
Supplement: Supplementary file 1 — Supplementary Material 1 [file 10554_2025_3519_MOESM1_ESM.docx]

**Supplementary Material**

**Journal:** The International Journal of Cardiovascular Imaging

**Article:** Myocardial fibrosis in severe asymptomatic versus symptomatic aortic stenosis: A cardiac magnetic resonance cross-sectional study

**Authors:** Katrine Aagaard Myhr^a^, Liv Andrés-Jensen^b^, Bjørn Strøier Larsen^c^, Charlotte Burup Kristensen^a^, Nana Køber^c^, Susanne Glasius Tischer^d^, Lars Lindholm Sørensen^d^, Søren Skøtt Schmiegelov^e^, Jesper James Linde^a^, Niels Grove Vejlstrup^a^, Jordi Sanchez Dahl^f^, Lars Køber^a^, Redi Pecini^a^

**Affiliations:**

^a^ Department of Cardiology, The Heart Centre, Copenhagen University Hospital – Rigshospitalet, Copenhagen, Denmark

^b^ Department of Pediatric Hematology/Oncology, Juliane Marie Centre, Copenhagen University Hospital – Rigshospitalet, Copenhagen, Denmark; Copenhagen Oncology Research Laboratory (Bonkolab), Juliane Marie Centre, Copenhagen University Hospital – Rigshospitalet, Copenhagen, Denmark

^c^ Department of Cardiology, Copenhagen University Hospital – Bispebjerg and Frederiksberg, Copenhagen, Denmark

^d^ Department of Cardiology, Copenhagen University Hospital – Amager and Hvidovre, Copenhagen, Denmark

^e^ Department of Cardiology, Zealand University Hospital, Roskilde, Denmark

^f^ Department of Cardiology, Odense University Hospital, Odense, Denmark

**Corresponding Author** Correspondence concerning this article should be addressed to Katrine A. Myhr. Email: katrine.aagaard.myhr.01@regionh.dk.

Table S1 Baseline cardiac magnetic resonance parameters and comparison of patients with asymptomatic severe aortic stenosis from the DANAVR trial (reference) (n=36) and symptomatic severe aortic stenosis from the FIBROTIC study (n=80) using multivariable linear and logistic regression analyses adjusted for sex, age, and peak aortic velocity

| **Parameter** | **Asymptomatic AS/DANAVR (n=36)** | **Symptomatic AS/FIBROTIC (n=80)** | **Comparison:**  **β or OR (95% CI)** | **p-value** |
| --- | --- | --- | --- | --- |
| **Left ventricular geometry and function** | | | | |
| LVEDVi, ml/m^2^ | 75±18 | 77±15 *[n=79]* | 0.3 (-6 to 6) | 0.92 |
| LVESVi, ml/m^2^ | 23±11 | 25±9 *[n=79]* | 3 (-1 to 7) | 0.19 |
| LVSVi, ml/m^2^ | 52±11 | 52±12 *[n=79]* | -2 (-7 to 2) | 0.33 |
| LVMi, g/m^2^ | 79±18 | 79±18 *[n=79]* | -4 (-11 to 2) | 0.21 |
| LV hypertrophy, n (%) | 15 (42) | 27 (34) *[n=79]* | 0.5 (0.2 to 1.2) | 0.13 |
| LVEF, % | 70±9 | 68±9 *[n=79]* | -4 (-8 to -0.5) | **0.03** |
| GLS, % | -17±2 *[n=35]* | -16±2 *[n=78]* | -0.5 (-1 to 0.5) | 0.32 |
| **Myocardial tissue characterization** | | | | |
| Native T1, ms | 1029±25 | 1028±26 | -2 (-13 to 8) | 0.65 |
| ECV%, % | 26±3 *[n=31]* | 26±2 *[n=67]* | 0.2 (-1 to 1) | 0.70 |
| LGE positive, n (%) | 13 (40) *[n=32]* | 28 (41) *[n=69]* | 0.1 (-1 to 1) | 0.83 |
| LGE pattern, n (%)  Ischemic  Non-ischemic | 0 (0)  13 (100) | 4 (14)  24 (86) | 0.19 (0.001 to 2.2) | 0.20 |
| LGE volume percentage, % | 2.1 (0.8-3.8) | 2.0 (0.8-3.5) | -0.4 (-2 to 1) | 0.52 |
| Myocardial fibrosis grade, n (%)^a^  High-grade  Low-grade | *[n=30]*  5 (20)  25 (80) | *[n=64]*  9 (14)  55 (86) | 0.90 (0.2 to 3.5) | 0.87 |

Baseline values are mean±SD, median (interquartile range), or count (percentage). In case of missing observations, the total number of observations is presented in square brackets. Linear and logistic regression analyses are presented with β-values or odds ratios and 95% confidence intervals, respectively. Asymptomatic patients were used as reference. Significant p-values are presented in bold.

ECV%, extracellular volume; GLS, global longitudinal strain; LGE, late gadolinium enhancement; LV hypertrophy, left ventricular hypertrophy; LVEDVi, left ventricular end-diastolic volume index; LVSVi, left ventricular end-systolic volume index; LVEF, left ventricular ejection fraction; LVMI, left ventricular mass index; LVSVi, left ventricular stroke-volume index.

^a^ Myocardial fibrosis grade: Native T1, ECV%, and LGE prevalence combined in a single high-grade or low-grade myocardial fibrosis parameter

Table S2 Baseline cardiac magnetic resonance parameters and comparison of patients with asymptomatic (reference) (n=42) and symptomatic (n=66) severe aortic stenosis using multivariable linear and logistic regression analyses adjusted for sex, age, and peak aortic velocity. Patients with ischemic late gadolinium enhancement were excluded.

| **Parameter** | **Asymptomatic AS (n=42)** | **Symptomatic AS (n=75)** | **Comparison:**  **β or OR (95% CI)** | **p-value** |
| --- | --- | --- | --- | --- |
| **Left ventricular geometry and function** | | | | |
| LVEDVi, ml/m^2^ | 7419 | 77±14 *[n=74]* | 1 (-5 to 6) | 0.86 |
| LVESVi, ml/m^2^ | 22±11 | 25±8 *[n=74]* | 2 (-1 to 6) | 0.19 |
| LVSVi, ml/m^2^ | 51±12 | 53±12 *[n=74]* | -2 (-6 to 3) | 0.41 |
| LVMi, g/m^2^ | 77±19 | 79±17 *[n=74]* | -3 (-9 to 4) | 0.40 |
| LV hypertrophy, n (%) | 17 (40) | 25 (34) *[n=74]* | 0.5 (0.2 to 1.3) | 0.16 |
| LVEF, % | 70±9 | 68±9 *[n=74]* | -4 (-7 to -0.4) | **0.03** |
| GLS, % | -17±2 *[n=41]* | 17±3 *[n=73]* | 1 (-0.3 to 1) | 0.20 |
| **Myocardial tissue characterization** | | | | |
| Native T1, ms | 1028±26 | 1029±27 | -0.4 (-11 to 10) | 0.94 |
| ECV%, % | 26±3 *[n=36]* | 26±2 *[n=63]* | 0.3 (-1 to 1) | 0.60 |
| LGE positive, n (%) | 14 (37) *[n=38]* | 23 (37) *[n=64]* | 1.0 (0.3 to 2.7) | 0.94 |
| LGE volume percentage, % | 2.1 (0.9-3.8) | 1.9 (0.8-3.5) | -0.5 (-2 to 1) | 0.50 |
| Myocardial fibrosis grade, n (%)^a^  High-grade  Low-grade | *[n=35]*  5 (14)  30 (86) | *[n=60]*  8 (13)  52 (87) | 1.0 (0.3 to 3.8) | 0.96 |

Baseline values are mean±SD, median (interquartile range), or count (percentage). In case of missing observations, the total number of observations is presented in square brackets. Linear and logistic regression analyses are presented with β-values or odds ratios and 95% confidence intervals, respectively. Asymptomatic patients were used as reference. Significant p-values are presented in bold.

ECV%, extracellular volume; GLS, global longitudinal strain; LGE, late gadolinium enhancement; LV hypertrophy, left ventricular hypertrophy; LVEDVi, left ventricular end-diastolic volume index; LVSVi, left ventricular end-systolic volume index; LVEF, left ventricular ejection fraction; LVMI, left ventricular mass index; LVSVi, left ventricular stroke-volume index.

^a^ Myocardial fibrosis grade: Native T1, ECV%, and LGE prevalence combined in a single high-grade or low-grade myocardial fibrosis parameter
